# MORPH: Probabilistic Alignment Combined with Hidden Markov Models of *cis*-Regulatory Modules

**DOI:** 10.1371/journal.pcbi.0030216

**Published:** 2007-11-09

**Authors:** Saurabh Sinha, Xin He

**Affiliations:** Department of Computer Science, University of Illinois at Urbana-Champaign, Urbana, Illinois, United States of America; Weizmann Institute of Science, Israel

## Abstract

The discovery and analysis of *cis-*regulatory modules (CRMs) in metazoan genomes is crucial for understanding the transcriptional control of development and many other biological processes. Cross-species sequence comparison holds much promise for improving computational prediction of CRMs, for elucidating their binding site composition, and for understanding how they evolve. Current methods for analyzing orthologous CRMs from multiple species rely upon sequence alignments produced by off-the-shelf alignment algorithms, which do not exploit the presence of binding sites in the sequences. We present here a unified probabilistic framework, called MORPH, that integrates the alignment task with binding site predictions, allowing more robust CRM analysis in two species. The framework sums over all possible alignments of two sequences, thus accounting for alignment ambiguities in a natural way. We perform extensive tests on orthologous CRMs from two moderately diverged species Drosophila melanogaster and D. mojavensis, to demonstrate the advantages of the new approach. We show that it can overcome certain computational artifacts of traditional alignment tools and provide a different, likely more accurate, picture of *cis-*regulatory evolution than that obtained from existing methods. The burgeoning field of *cis-*regulatory evolution, which is amply supported by the availability of many related genomes, is currently thwarted by the lack of accurate alignments of regulatory regions. Our work will fill in this void and enable more reliable analysis of CRM evolution.

## Author Summary

Interspecies comparison of regulatory sequences is a major focus in the bioinformatics community today. There is extensive ongoing effort toward measuring the extent and patterns of binding site turnover in *cis-*regulatory modules. A major roadblock in such an analysis has been the fact that traditional alignment methods are not very accurate for regulatory sequences. This is partly because the alignment is performed independently from the binding site predictions and turnover analysis. This article describes a new computational method to compare and align two orthologous regulatory sequences. It uses a unified probabilistic framework to perform alignment and binding site prediction simultaneously, rather than one after the other. Predictions of binding sites and their evolutionary relationships are obtained after summing over all possible alignments, making them robust to alignment ambiguities. The method can also be used to predict new *cis-*regulatory modules. The article presents extensive applications of the method on synthetic as well as real data. These include the analysis of over 200 *cis-*regulatory modules in D. melanogaster and their orthologs in D. mojavensis. This analysis reveals a significantly greater degree of conservation of binding sites between these two species than will be inferred from existing alignment tools.

## Introduction

Two-sequence alignment has been an indispensable tool in the bioinformatician's repertoire for nearly two decades now [1,2]. With scientific interest swinging toward the noncoding part of the genome, there has been a recent upsurge in adapting alignment algorithms beyond the usual tasks of identifying gene or protein orthologs. In the absence of the relatively rigid organization of coding sequences, noncoding sequences are often hard to align over moderate evolutionary divergences. Even in cases in which sequence homology is established on the scale of, say, a few hundred base pairs, the actual alignment of these orthologous noncoding sequences is ambiguous. This in turn impedes comparative analysis of *cis-*regulatory sequences, which relies on an accurate knowledge of base-level orthology. The natural response to this challenge has been the proposal of probabilistic alignment methods that can, for example, provide a confidence value on any two bases being aligned (orthologous) without committing to any single “best” alignment. We shall see below some of the successful manifestations of this idea.

A somewhat orthogonal line of research with respect to noncoding sequence analysis has been the search for *cis-*regulatory modules or CRMs (sometimes called enhancers) by scanning for statistically significant clusters of transcription factor binding sites, which in turn are detected by sequence similarity to a priori known “motifs.” Discovery of CRMs has played a key role in understanding gene regulation in metazoa, especially the fruitfly [3] and the sea urchin [4]. Although the earliest genome-wide computational scans for CRMs were based on counting high-quality matches to the motifs [5,6], it was not long before probabilistic approaches permeated this area, and efficient implementations of Hidden Markov models (HMMs) led to CRM discovery with high sensitivity [7,8]. The application of HMMs to CRMs allows us to consider all possible ways of “parsing” a CRM as a collection of binding sites interspersed with random bases, while weighting each parse by a probabilistic score. It was shown previously [9] how the HMM framework can be integrated with multispecies comparison, in an algorithm called “Stubb,” in order to improve CRM discovery. This is achieved by using sequence alignment as a first step, and modeling aligned binding sites by a stochastic model of binding site evolution. One limitation of this algorithm is that it assumes that the correct alignment can be computed (e.g., by using the alignment program LAGAN [10]) in its first step.

Here, we combine the two seemingly separate ideas mentioned above—probabilistic alignment of two sequences and probabilistic analysis of CRMs—into an integrated probabilistic framework. The new framework provides a robust way to compare CRMs across moderate evolutionary distances at which sequence-level regulatory changes are prominent and tractable. We implement efficient procedures for learning the parameters of the model, based on the expectation-maximization (E-M) technique. Two programs with related but somewhat distinct functionalities are made available: (1) the “MorphMS” program predicts CRMs located within a pair of orthologous sequences, whereas (2) the Morphalign program constructs an alignment of two given sequences and uses a novel display format to point out the ambiguities in the alignment as well as the locations of putative binding sites. Both programs require as input a set of transcription factor binding motifs that the user is interested in. All parameters of the model, except for the length of CRMs to be predicted by MorphMS, can be automatically learned from the data. The alignments produced by the Morphalign program are viewable in the highly portable HTML format, and both MorphMS and Morphalign are available for download as source code (see Supplementary Materials at our site http://veda.cs.uiuc.edu/Morphalign/supplement/ (http://rd.plos.org. pcbi_0030216_0001).

We first use synthetic data to demonstrate that the new probabilistic model, henceforth called the MORPH framework, can lead to highly significant improvement in (1) alignment accuracy on *cis-*regulatory sequences, as compared to a state-of-the-art alignment program, and (2) binding site prediction accuracy, as compared to an HMM-based program (Stubb [9]) that works with a fixed alignment. We next apply the framework to a comprehensive collection of CRMs in two species of fruitfly—D. melanogaster and D. mojavensis—and present our alignments and binding site predictions through a Web interface. We demonstrate a remarkable improvement in CRM prediction accuracy, for this dataset, over that from the HMM-based Stubb program. We find that probabilistically summing over all possible alignments and using binding sites during alignment provide a very different picture of orthologous CRM relationships than existing approaches. We show that this greatly affects the conclusions one draws about binding site loss and gain between species. We expect the MORPH framework to strongly impact future studies on *cis-*regulatory evolution and binding site turnover.

### Previous Work

Sequence alignment is an intensely researched topic with several major achievements, and we refer the reader to [11] for a review of this field. The highly popular, scoring function–based alignment method of Needleman and Wunsch [1] has a natural extension to probabilistic methods, as shown by Holmes and Durbin [12]. They modeled alignment generation as a first-order Markov process involving three states called “Match,” “Insert,” and “Delete,” with the Match state generating aligned pairs of bases and the latter two states emitting gap-aligned bases. This type of model, called pair-HMM, has been used in a number of studies, with differences in the model details. For example, the ProbCons algorithm of Do et al. [13] uses the same Match, Insert, and Delete states as in [12,14], but does not allow any direct transitions between Insert and Delete states.

Another class of probabilistic methods, called statistical alignment, uses an evolutionarily motivated stochastic process of indels (insertions and deletions) to construct the maximum-likelihood alignment. Earlier work in this class, including TKF91 model [15] and its equivalent HMM formulation [16], is based on a simple indel process, where at each position, a single nucleotide is randomly inserted or deleted following a Poisson process. Later work improves the model by allowing insertions or deletions of multiple nucleotides as a single event, and a pair-HMM approximation of this complicated stochastic process has been used in [17,18]. The main advantage of the pair-HMM and/or statistical alignment methods is that the parameters—the transition probabilities, indel length distribution parameter (often assumed to be geometric), and sometimes the nucleotide emission probabilities—can all be estimated automatically from the input sequences, using maximum likelihood method, without external training. We borrow the pair-HMM framework in the alignment model of MORPH.

Our previously published probabilistic method called Stubb [9] comes closest to how the MORPH framework deals with *cis-*regulatory sequences from two species. As mentioned above, Stubb first finds the optimal scoring alignment using a standard alignment program such as LAGAN [10], and fixes this alignment. It then uses a probabilistic model that generates orthologous CRMs by transitioning among “motif” and “background” states, and sampling binding sites or “background” nucleotides (respectively) from appropriate emission probability distributions. When generating aligned positions, the orthology of binding sites is modeled using a simple stochastic model parameterized by the known sequence specificity (motif) of the binding sites. The MORPH framework uses exactly the same model for generating orthologous CRMs, except that the alignment is not fixed in the first step, and is modeled probabilistically as explained in the previous paragraphs.

One of the first attempts to couple alignment with binding site predictions was made in the program CONREAL [19]. This program predicts binding sites from a given set of position weight matrices (PWMs), and uses pairs of conserved binding sites to serve as anchors in a traditional sequence alignment algorithm. Recently, a method called EEL [20] has been proposed to predict *cis-*regulatory sequences while constructing sequence alignment at the same time, an objective that overlaps with ours. It first scans the given pair of orthologous sequences to find all putative binding sites, and then applies the Smith-Waterman dynamic programming algorithm to the sequences of binding sites. That is, the binding sites are the basic units (symbols) to be aligned, not nucleotides. The alignment parameters, in particular the penalty for changed spacing between two adjacent pairs of aligned binding sites, are estimated from external data. Our probabilistic framework MORPH offers several important advantages over such a method: (1) the uncertainty in assigning binding sites is handled by using a probabilistic model for CRM, so no cutoff is needed for determining binding sites; (2) the parameters in the model are automatically estimated; and (3) the ambiguity in alignment as well as binding site annotation can be precisely quantified using probabilities (see below). Additionally, MORPH simultaneously considers both background sequences and binding sites for alignment, unlike EEL, which ignores the non-site sequences.

## Results/Discussion

### Model

We begin by describing the probabilistic process (the “MORPH” model) that generates two orthologous sequences (CRMs), given a set of transcription factor binding motifs in the form of their PWMs [21]. A PWM specifies the probability distribution of nucleotides at each position of the binding site and is typically determined from multiple alignments of experimentally characterized binding sites. Motif databases such as TRANSFAC [22], JASPAR [23], and FlyReg [24] also provide such PWMs.

Here, we provide an informal description of the MORPH model, and leave the detailed description for Materials and Methods. At the outer level of the model, we use a first-order Markov process with three states: Match, Delete, and Insert. The Match state corresponds to aligned positions and emits two equal-length strings (one for each species). The Delete and Insert states correspond to unaligned positions in either species and emit a string that will be appended to the first or second species' sequence, respectively. Thus, this outer level of the model generates alignments with blocks of aligned positions separated by unaligned strings in either or both species.

The string emitted in any of the three states of the above process is chosen by another probabilistic process, which models the interspersed arrangement of binding sites and non-binding “background” sequence in the CRMs. This process first chooses a particular motif and samples a string from the probability distribution prescribed by that motif (PWM). In the Match state, the sampled string is “evolved” using an evolutionary model to obtain two related strings that are then emitted. The motif choices available include all input PWMs as well as a single-column “background” PWM that models random sequence.

The model parameters include all transition probabilities among the Match, Insert, and Delete states, as well as the transition probabilities into each motif state. These parameters are trained using an E-M strategy employing dynamic programming for efficient calculations. For the two evolutionarily related strings emitted from a Match state, there is a model parameter representing evolutionary divergence of the species. This is either user-specified (e.g., by using the PAML package [25] to estimate neutral substitution probabilities), or it can be automatically learned from the data.

A note regarding the semantics of the alignment generating process is in order. In previous work on probabilistic alignment, such as Holmes and Durbin [12], all transitions (among the three states Match, Insert, and Delete) are allowed, except for Insert → Delete. The pairwise alignment is viewed here as comprising (1) blocks of successive aligned positions and (2) unaligned sequences in both species separating them. For the unaligned sequences between any two blocks, there is no notion of the order in which they were generated in the two species. Hence, we may arbitrarily assume that the entire interblock sequence in the first species was generated first, followed by the entire interblock sequence in the second species. This is represented by a Delete → Insert state transition. The same semantics of unaligned sequences are adopted in the MORPH framework.

### Experiments with Synthetic Data

We first performed extensive experiments with synthetic datasets, where “orthologous CRMs” were generated artificially. Testing on synthetic data has become a common practice in evaluation of bioinformatics algorithms today, offering the following advantages: (1) the correct answers are known in synthetic data, (2) the datasets are created with complete control over different aspects of the signal strength, and (3) large numbers of datasets can be obtained. Therefore, synthetic datasets allow us to evaluate and compare various algorithms, and gain insights into how such comparisons depend on different aspects of the data. Here, we obtained synthetic “orthologous CRMs” by sampling from the MORPH probabilistic model. A set of seven PWMs, corresponding to transcription factors involved in early development in *Drosophila*, were included in the model, and their motif transition probabilities were set to *p_i_* = 0.01. The parameters μ_I_ and μ_D_, which determine the length distribution of unaligned sequences (Materials and Methods), were set to be equal and to range between 0.2 and 0.8, in increments of 0.1. The parameter μ, which is related to the length distribution of aligned blocks, was varied between 0.05 and 0.20, in increments of 0.05. For each combination of μ_I_ (μ_D_) and μ, called an “experiment set,” we obtained ten pairs of sequences.

#### Improvement in alignment accuracy.

**Figure 1:**
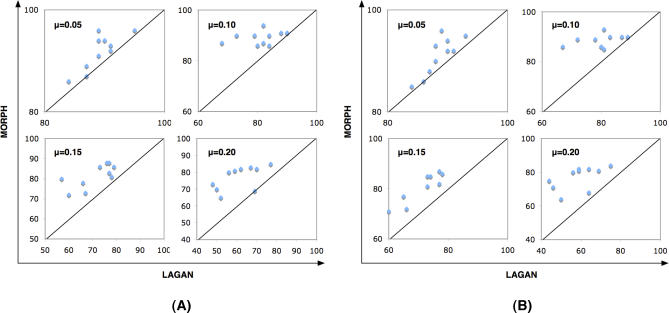
Alignment Sensitivity (A) and Specificity (B) of LAGAN and Morphalign (A) Sensitivity and (B) specificity of LAGAN and Morphalign on experiment sets with simulation parameters μ_I_ = μ_D_ = 0.2 are shown. Diagonal lines represent equal scores.

In the first analysis, we evaluated the accuracy of computed alignments by comparing them with the true alignments. For each of the ten sequence pairs in an experiment set, we counted what percentage of the truly aligned positions are aligned by the computed alignment, thus obtaining an “alignment sensitivity” that ranges between 0% (worst) to 100% (best). An “alignment specificity” was similarly computed. These two scores were computed for the popular alignment tool LAGAN, as well as for the Morphalign program, with PWMs and the *φ* (evolutionary divergence) parameter being known and all other parameters learned from the data. The Morphalign program computes a global alignment of two sequences using the MORPH framework. In this exercise, we configured it to report the maximum likelihood (Viterbi) alignment (see “Morphalign” in Materials and Methods). The alignment parameters (μ_I_, μ_D_, and μ) and motif transition probabilities (*p_i_*) are automatically learned by Morphalign for each sequence pair. Figure 1 compares the alignment sensitivity and specificity of these two alignment methods for all sequence pairs in experiment sets having μ_I_ = μ_D_ = 0.2, and different values of μ. We see a clear and consistent improvement in alignment accuracy (both sensitivity and specificity) when using Morphalign (and PWM knowledge), compared to LAGAN, with the improvement being more pronounced as the aligned blocks get shorter (larger μ). The same trend is seen for other values of μ_I_ ( = μ_D_) (unpublished data). This test therefore shows us how we may get more accurate alignments of orthologous regulatory sequences if we know the relevant binding site motifs and exploit them within the MORPH framework. We also repeated these tests on synthetic datasets generated by a simple evolutionary model that is different from the MORPH probabilistic model. We find again, as shown in the Supplementary Materials at ((http://rd.plos.org. pcbi_0030216_0001), that the Morphalign program provides significantly greater sensitivity and specificity of alignments than LAGAN run with default parameters. Details of the new simulation program, which is based on the Dawg program [26], are also are provided in the Supplementary Materials at (http://rd.plos.org. pcbi_0030216_0001).

#### Improvement in binding site prediction.

**Figure 2:**
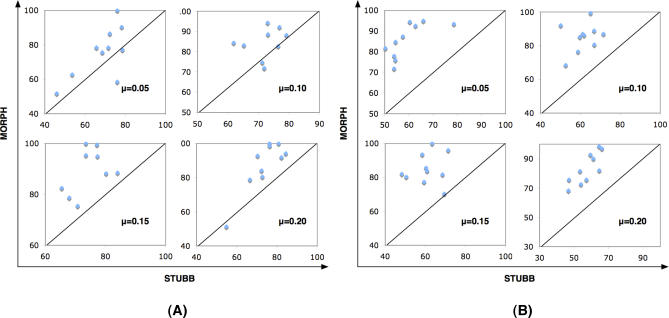
Binding Site Prediction Sensitivity (A) and Specificity (B) with Stubb and Morphalign (A) Sensitivity and (B) specificity with Stubb and Morphalign on experiment sets with simulation parameters μ_I_ = μ_D_ = 0.2 are shown.

In the second analysis, we investigated whether use of the probabilistic model of alignment improves binding site prediction over that from using a fixed alignment, as in Stubb [9]. Given the true locations of the binding sites, and the predicted locations from an algorithm, it is straightforward to compute the nucleotide-level sensitivity and specificity of predictions. (This computation was done based on sites in only one of the “species”.) As in the previous section, these scores were computed for each of the ten sequence pairs in an experiment set. We compared the performance of Morphalign with that of Stubb (in the two-species mode)—both algorithms were given all known PWMs and thus are run with the same prior knowledge; whereas Stubb uses a preprocessing alignment step (based on LAGAN), Morphalign considers all possible alignments probabilistically. Each algorithm was made to predict locations of binding sites using the same confidence value (“marginal probability” threshold; see “Synthetic Data Experiments” in Materials and Methods). Figure 2 shows the sensitivity (2A) and specificity (2B) with both methods for all sequence pairs in experiment sets having μ_I_ = μ_D_ = 0.2 and different values of μ. We find a clear and consistent improvement in binding site prediction using Morphalign, in terms of both sensitivity and specificity. Morphalign's specificity is always significantly better, and so is its sensitivity. These results remain practically unchanged for other values of μ_I_ ( = μ_D_) (unpublished data). This analysis provides a compelling demonstration of the advantage of using the Morphalign program for binding site prediction from orthologous CRMs.

### Comparative Analysis of D. melanogaster and D. mojavensis CRMs

The recent sequencing of 12 *Drosophila* genomes (http://rana.lbl.gov/drosophila/) and the recent publication of the largest database of experimentally validated CRMs (REDfly [27]) has opened up the opportunity for us to examine orthologous regulatory sequences for their binding site content and mutual similarity. We chose to apply the MORPH framework on D. melanogaster and D. mojavensis, the latter being one of the most-diverged species (from the former) among the newly sequenced *Drosophila* genomes. These two species are expected to exhibit common modes of *cis-*regulation, using highly conserved transcription factors and DNA binding affinities (“motifs”). At the same time, they are diverged enough (∼40 Myr) to demonstrate substantial evolutionary flux at the binding site level. (See, for example, the works of Moses et al. [28] and Emberly et al. [29], both of which studied species with less divergence.) At the nucleotide level, the orthologous sequences we analyzed had a median percent identity of 52 (using LAGAN with default parameters). High incidence of short tandem repeats, including tandem repeats of binding sites, has been recorded in *Drosophila* CRMs [30], and is likely to create alignment ambiguities between the two species compared. For all these reasons, application of our MORPH framework to orthologous CRMs in D. melanogaster and D. mojavensis holds the promise of bringing out interesting evolutionary analyses of *cis-*regulation.

#### Computational prediction of a large class of CRMs.

We collected a set of 208 experimentally verified D. melanogaster CRMs from the REDfly database, spanning a broad spectrum of biological processes. For each CRM, we included 1-Kbp flanking sequence on either side, and extracted the ortholog of this entire sequence from D. mojavensis. (These sequences had a mean length of 2,702 bp in D. melanogaster, with a standard deviation of 535.) We also collected a set of 53 PWMs from FlyReg [24]. Our goal was (1) to align these orthologous CRMs and predict binding sites, orthologous or otherwise, using the MORPH framework, and (2) to investigate whether the MORPH framework is able to predict CRMs by using a fairly broad collection of known motifs.

It is expected that any given CRM is regulated by a small subset of the 53 motifs, but such information is not available for most of the CRMs. Therefore, we decided to use all 53 motifs in our initial analysis. As such, false positives are inevitable during binding site prediction, and more specific information about transcription factors (such as genetic information on transcription factor–gene regulatory interactions) is necessary to deal with these. A researcher interested in analyzing a specific class of CRMs, e.g., those involved in anterior–posterior patterning of the embryo (e.g., Schroeder et al. [3]), will use motifs corresponding to transcription factors known to regulate that class, and can expect to see significantly better predictions of binding sites. We come back to this issue in “Alignment and Regulatory Evolution in Two Specific Pathways” in Results/Discussion.

**Figure 3:**
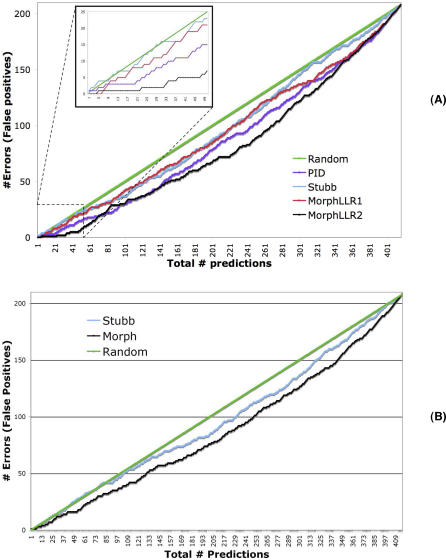
CRM Prediction Accuracy on a Dataset with 208 “Positive” Sequences (CRMs) and 208 “Negative” Sequences (Random Noncoding Genomic Fragments) The *y*-axis shows the number of negatives included in a given number (*x*-axis) of top scoring sequences. Green line (diagonal) represents error rate expected by chance. (A) Predictions were made based on percentage identity (PID, purple line), Stubb score (blue), Morph LLR1 score (red), and Morph LLR2 score (black). (B) Predictions were based on the total number of binding sites predicted by Stubb (blue) and Morph (black).

Our first exercise was to apply the MorphMS program on each CRM sequence. This program (see “Algorithm and Implementation” in Materials and Methods) slides a window of length 500 bp (in shifts of 250 bp) on the input sequence, and, for each position of this sliding window, reports two log-likelihood ratio (LLR) scores. The LLR1 score compares the likelihood (of the sequence data) under the MORPH model to the likelihood under a null model in which only the background PWM is used. The LLR2 score uses a null model in which the Match state is not allowed, which means that the two orthologous sequences are assumed to be generated independently. To examine whether the LLR1 and LLR2 scores are able to discriminate CRMs, we considered all 208 CRMs as the “positive” class of sequences, and collected equally many length-matched sequences (and their orthologs) from randomly selected noncoding regions of the genome, forming the “negative” class. We then asked how well the positive class can be discriminated from the negative class, based on these scores. In order to test the advantage offered by the MORPH framework, we also used the LLR1 score computed by the Stubb program. We chose to compare MORPH performance with Stubb because (1) Stubb's probabilistic framework is very similar to MORPH, except that Stubb relies on a “hard” alignment, and (2) Stubb has been shown to improve CRM prediction using two fruitfly genomes over its single-species version [31]. We also included in our comparisons the simple strategy of using the percentage identity (PID) between orthologous pairs of sequences as a classifier between the positive and negative classes. Each strategy (PID, Stubb, MorphMS LLR1, or MorphMS LLR2) was used to score all sequences, and the number of “errors” (i.e., negative-class sequences) included in the top *K* scoring sequences was plotted as a function of *K* (Figure 3A). Thus, the *y*-axis in Figure 3A is proportional to the false-positive error rate. The error rate expected by chance (50%) is shown as a reference. We find that the MorphMS LLR2 score provides significantly better discrimination than all other scores. For example, in the top 50 predictions by LLR2 score, there are only seven negative sequences and 43 positives. (The second-best strategy in this range is PID, which reports 15 negative sequences, more than double the error rate of LLR2.) This remarkable ability of the MorphMS LLR2 score to discriminate regulatory sequences is even more significant when we consider that our dataset includes CRMs from a wide spectrum of biological processes, and not just the blastoderm-stage embryonic segmentation pathway that has been the focus of previous computational studies [5,8,32,33]. This exercise also reveals that conservation information (LLR2) is substantially more effective than information on binding site clustering alone (LLR1 and Stubb) in our test scenario. In fact, even the simple strategy of computing PID gives better discrimination than LLR1 and Stubb. We believe that these two methods (LLR1 and Stubb) are not able to better distinguish CRMs from random sequences because they used an extremely broad collection of motifs, and should provide much improved results with small, pathway-specific sets of motifs. The LLR2 score, on the other hand, is guided by the increased conservation levels and not just the motif clustering, and hence performs significantly better. The improvement it brings over the PID score shows that it helps to measure conservation at the binding site level than at the raw nucleotide level.

Next, we investigated the accuracy of binding site predictions. Our synthetic data results have shown how MORPH can predict binding sites with added sensitivity and specificity, in comparison to Stubb. In the current dataset, MorphMS predicted many more sites than Stubb (unpublished data). However, it was not possible to directly assess which of the methods is more accurate, since far too few binding sites have been experimentally identified in these 208 CRMs. Nevertheless, we can get a high-level insight into performance by framing the evaluation in a classification setting, as follows. The idea is that the “better method” will predict more sites in CRMs compared to random genomic segments, to the extent that this differential prediction of binding sites may allow us to classify CRMs from random sequences. Hence, we considered the total number of binding sites predicted by each method in each sequence, and tested which method is better at discriminating the positive class (CRMs) from the negative class (random genomic sequences) based on these total site counts. We note that this is only an indirect way to assess binding site prediction accuracy, and does not allow us to evaluate individual site predictions. Figure 3B shows the false-positive rate (as in the previous paragraph) when using total site counts as the discriminating feature. First, both MorphMS and Stubb show significant departure from the random expectation. Moreover, MorphMS is consistently better than Stubb in terms of the error rate, presenting indirect evidence that its binding site predictions are more accurate. If we believe this evidence, it would seem that proper treatment of alignment ambiguities reveals many more binding sites than Stubb's hard alignment approach, a conclusion that will be further corroborated in the next section.

### Alignment and Regulatory Evolution in Two Specific Pathways

The above analysis was performed on CRMs spanning many different pathways, using a very broad collection of motifs, to get a relatively unbiased view of MORPH performance. We next show results from two specific developmental pathways. We collected two sets of CRMs from REDfly: “BLASTODERM.A-P,” a set of 54 CRMs regulating anterior–posterior segmentation at the blastoderm stage, and “MESODERM,” a set of 46 CRMs involved in mesoderm specification. For each set, we also collected a set of motifs (PWMs) for transcription factors believed to be involved in that pathway—a collection of ten and 18 motifs for the BLASTODERM.A-P and MESODERM sets, respectively. (See Supplementary Materials at http://rd.plos.org. pcbi_0030216_0001 for lists of these CRMs and motifs.) For each dataset, the Morphalign program (Materials and Methods) was used to align and predict binding sites in the D. melanogaster and D. mojavensis orthologs of each CRM. (The alignments were displayed using the maximum expected accuracy alignment as backbone; see “Morphalign” in Materials and Methods.) We analyzed the results of this exercise with respect to insights it provides about evolution of CRMs, and how such insights differ from traditional methods of evolutionary sequence comparison. The complete set of alignments for both sets is available online (see Supplementary Materials at http://rd.plos.org. pcbi_0030216_0001), and will be a valuable resource for biologists studying the evolution of these important sets of CRMs, or of CRMs in general.

#### Morphalign highlights ambiguities in alignment.

**Figure 4:**
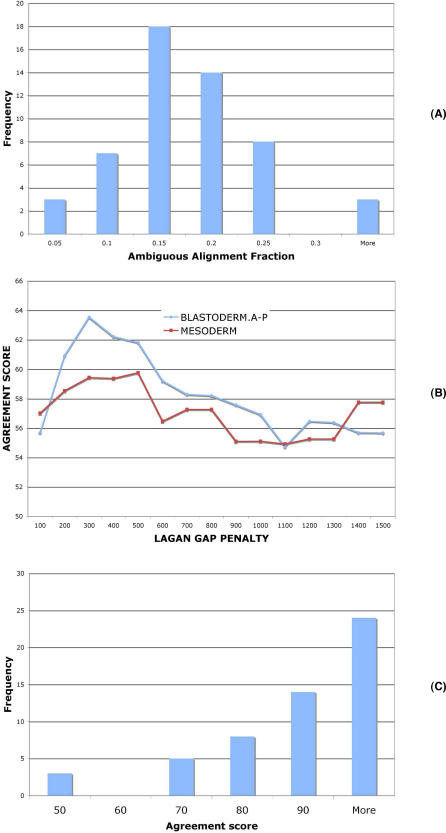
Morphalign Alignments: Differences from Alternative Alignment Methods (A) For each CRM in the BLASTODERM.A-P set, the fraction of the D. melanogaster sequence that was ambiguously aligned (to two or more positions in D. mojavensis) was computed. The figure shows the histogram of these alignment ambiguity fractions for the set. (B) Median of alignment agreement scores between output of Morphalign and output of LAGAN (run separately with a range of gap opening penalties) for CRMs in the BLASTODERM.A-P (blue) and the MESODERM (red) sets. (C) Histogram of alignment agreement scores between Morphalign and its no-motifs version, for the BLASTODERM.A-P set of CRMs.

A distinct difference of Morphalign alignments from traditional (non-probabilistic) alignment programs is that ambiguities in the alignment are explicitly pointed out. To quantify this aspect, we asked what fraction of the positions in one species (D. melanogaster) were aligned with marginal probability above a threshold of 0.1 to two or more positions in the second species (D. mojavensis). This measure, which we call the alignment ambiguity fraction, is shown in Figure 4A. We find the majority of the BLASTODERM.A-P CRMs to have between 10% and 20% ambiguous positions by this measure. One obvious source of alignment ambiguities is the presence of short tandem repeats in the CRMs, whose high frequency has been reported in [30]. We computed the separations between positions that are aligned to the same position in the other species, and found the median separation to be very small (6 bp for BLASTODERM.A-P and 4 bp for MESODERM), i.e., most ambiguities are “local.” We also computed statistical significance of overlaps between ambiguously aligned positions (the two or more positions in D. mojavensis aligned to the same position in D. melanogaster) and tandem repeat positions of the CRMs (predicted using TRF, the Tandem Repeat Finder program [34]), using a hypergeometric test (*p* < 0.01). Of the 43 CRMs in BLASTODERM.A-P that had over 10% alignment ambiguity, 14 CRMs had a significant overlap of ambiguously aligned and tandem repeat positions. Similar results were found in the MESODERM set. This provides statistical evidence that short tandem repeats play a large role in creating alignment ambiguities in CRMs.

Although the above observation points to short repeats as a source of alignment ambiguity, we also found ambiguously aligned positions separated by relatively large distances within the CRM. (That is, position *a* in one species aligned to two different positions *b* and *c* in the second species, with *b* and *c* being separated by a large distance.) For this, we looked at contiguous stretches of ten or more ambiguously aligned positions (in one species), and measured the distance between their two aligned regions in the other species. Twenty percent of such cases had separation more than 10 bp (i.e., subsequence *a* aligned to subsequences *b* and *c* that are separated by more than 10 bp). This suggests that these alignment ambiguities are the result of mechanisms other than tandem repeats, or that insertions have happened since the tandem repeat was created that have significantly separated the alternative alignable regions.

#### Morphalign alignments are different from conventionally obtained alignments.

LAGAN [10] is a popular tool for pairwise sequence alignment, and was therefore a natural choice with which to compare the Morphalign alignments. We first note that LAGAN alignments crucially depend on the user-specified “gap penalty” parameters, whereas Morphalign has no such parameters and automatically learns the “best” parameter values to use. We therefore compared the alignments from Morphalign to those from running LAGAN with different values of the gap opening penalty. The “Agreement score” used to compare two alignments is defined as the number of positions of the first species that are identically aligned in both alignments. As seen in Figure 4B, the LAGAN alignments are very different from Morphalign alignments (median agreement score less than 65% for both datasets, across the board). This demonstrates that using a probabilistic framework for pairwise alignment, along with binding site predictions, gives a very different picture of sequence similarities from that obtained using a traditional alignment tool such as LAGAN, regardless of the gap parameters.

**Figure 5:**
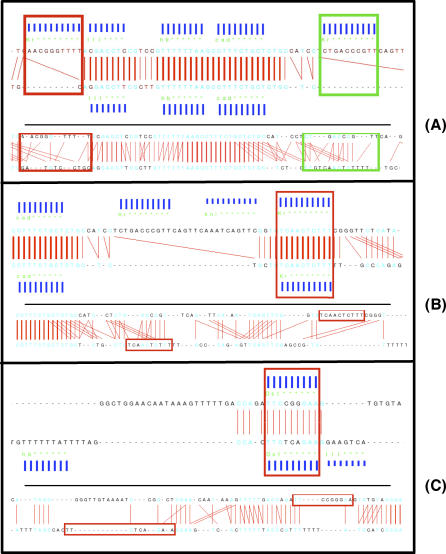
Examples of Difference in Alignments Produced by Morphalign and Its No-Motifs Version, for the hairy stripe 6 enhancer Each panel shows one example, with the Morphalign alignment at the top of that panel and the motif-agnostic alignment at the bottom. D. melanogaster is shown as the top sequence in an alignment. Vertically aligned positions are shown in blue if they are identical, in black otherwise. Red lines indicate aligned positions, with their thickness proportional to confidence (marginal probability) of that positional alignment. Only positional alignments with confidence greater than 0.1 are marked by red lines. (Note that one position may align with multiple positions in the other alignment.) Morphalign additionally shows predicted binding site locations with blue bars (whose height represents confidence level), and the motif names in green characters. (A) The red boxes show a predicted Kruppel site that is entirely unaligned by Morphalign, but is poorly and ambiguously aligned by the motif-agnostic alignment. The green box shows a similar situation. (B) A very well-aligned block with conserved Kruppel sites is found by Morphalign, but the sites are clearly separated in the motif-agnostic alignment. (C) A DStat site is aligned between the two species (by Morphalign). The no-motifs alignment conspicuously separates these potentially orthologous sites.

We also compared the Morphalign alignments to those obtained by running the same program without any motifs, i.e., a probabilistic alignment program that does not predict binding sites in the process of alignment. This differs from LAGAN in its probabilistic nature and the fact that alignment parameters are learned automatically from the data. Figure 4C shows the histogram of agreement scores between these two alignment methods for CRMs of the BLASTODERM.A-P set. (Similar results were obtained for the MESODERM dataset; unpublished data.) Majority of the CRMs show high alignment agreement (46/54 above 70%), whereas three (hairy stripe 7, hairy stripe 6, and slp1 A) show less than 50% agreement. The latter are of particular interest to us, since these are where using binding site predictions made the greatest difference to the alignments. We scrutinized the hairy stripe 6 CRM to find the source of poor agreement (42%) between Morphalign and its “no-motifs” version. Figure 5 shows several interesting differences between the alignments. Figure 5A shows two binding sites of Kruppel (red and green boxes) that are present in D. melanogaster and not in D. mojavensis, and are unaligned in the Morphalign alignment (top), but have been aligned with several gaps in the no-motifs version. Figure 5B shows a highly conserved binding site for Kruppel (red box) being aligned by the former method, but unaligned or erroneously aligned by the latter method. Figure 5C shows a strong match to the DStat motif (red box) in D. melanogaster being aligned to a weaker match in D. mojavensis, as per the Morphalign program. These seemingly orthologous DStat sites are not aligned by the no-motifs version of the program. Thus, manual inspection suggests that Morphalign, using motif information, is able to identify (potentially) orthologous binding sites even when its no-motifs version cannot.

#### Morphalign presents a different picture of binding site loss and gain.

A question that many researchers are interested in is the evolutionary dynamics of binding sites in CRMs: to what extent are binding sites conserved between species, and how do their evolutionary rates compare to various “neutral” rates? An indispensable component of such studies is the alignment of orthologous CRMs, to find conserved and non-conserved binding sites. Typically, this step is done using traditional alignment programs such as LAGAN, which is acknowledged to be a weak link in the analysis, and is sometimes supplemented with a manually performed realignment step. Morphalign, with its integrated view of binding site prediction and evolutionary events (indels and substitutions), is therefore likely to help produce a different and perhaps more accurate picture of binding site evolution. Here, we present evidence that the picture presented by Morphalign alignments is indeed very different from what LAGAN alignments suggest.

**Figure 6:**
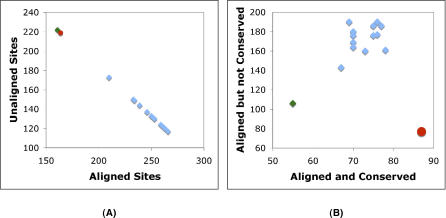
A Total of 383 Binding Sites for the Bicoid Transcription Factor Were Predicted Computationally (Using a Threshold) in D. melanogaster CRMs of the BLASTODERM.A-P Dataset (A) The number of sites that were aligned, versus the number of unaligned sites, using different alignments: Diamonds = LAGAN with varying gap penalties; red circle = Morphalign. (Green diamond represents LAGAN with lowest gap penalty.) (B) Of the sites aligned by a method, how many were conserved (i.e., PWM match score above threshold) in D. mojavensis. Color code is as in (A).

We used a well-characterized PWM of the Bicoid transcription factor, predicted all binding sites above a threshold in the D. melanogaster BLASTODERM.A-P set, and asked how many of these sites were (1) aligned to a D. mojavensis site also above the PWM match threshold (see “D. melanogaster and D. mojavensis Comparisons” in Materials and Methods), (2) aligned to a site below threshold, and (3) unaligned. We examined these numbers using Morphalign alignment, as well as with LAGAN alignments that used a wide range of gap penalties. There is an expected tradeoff between sensitivity and specificity here: LAGAN alignments with a high gap penalty will tend to produce more aligned regions, and hence align more binding sites (of D. melanogaster), but at the cost of aligning sites that are not “truly” orthologous. One way to control for this is to compare alignments that overall align similar fractions of the CRMs (or numbers of binding sites), and then check what fraction of the aligned binding sites are conserved (i.e., match PWM above threshold) in the second species. If we assume that most alignable binding sites are functional in both species (i.e., infrequency of lineage-specific selection), the better alignment should have more of its aligned binding sites conserved. Indeed, Figure 6A shows that all but one of the LAGAN runs (blue diamonds) differ substantially from Morphalign in terms of the fraction of D. melanogaster sites aligned. Only the LAGAN run with the lowest gap penalty (green diamond) agreed with Morphalign (red circle) with respect to this measurement. However, as Figure 6B shows, this run of LAGAN (green diamond) was significantly different from Morphalign (red circle) in terms of the fraction of aligned sites that were above threshold in both species. Both programs align a total of approximately 160 sites (Figure 6A), but Morphalign aligned 87 of these sites with potential sites in D. mojavensis, whereas LAGAN (with lowest gap penalty) had only 55 of the 160 aligned sites conserved in the second species, suggesting a much higher rate of binding site loss or gain. Another way to look at these results is simply to count what fraction of the sites aligned by a program were conserved: although Morphalign finds this fraction to be 53%, the LAGAN runs across the spectrum of gap penalties projected a value of 28%–34% (data inferred from Figure 6A and 6B). Thus we find, as expected, that Morphalign has a stronger tendency to align conserved binding sites in the two species, as compared to traditional, motif-agnostic alignment tools like LAGAN. Studies on *cis-*regulatory evolution, which often focus on the aligned binding sites in multiple species, will therefore report very different findings when using Morphalign and LAGAN, respectively. Similar results were obtained with well-characterized PWMs for other transcription factors such as Kruppel, Hunchback, Caudal, Tailless, and DStat (unpublished data).

**Figure 7:**
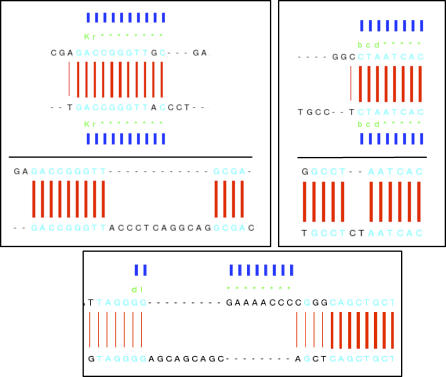
Snapshots from Alignments of Three CRMs: eve stripe 2 (Top Left), eve stripe1 (Top Right), and stumps_hbr_early (Bottom) In the top two panels, the alignment above was produced by Morphalign, and the alignment below it was produced by LAGAN (with gap opening penalty of 300).

Having shown that Morphalign gives a different picture of binding site evolution, we investigated whether this is justified. We therefore analyzed some specific examples and found that the difference is in part due to the ability of Morphalign to overcome some alignment errors. Figure 7 (top left) shows the output of Morphalign on a portion of the eve stripe 2 enhancer [27], with a pair of predicted Kruppel sites aligned to each other. (Kruppel is a known regulator of this CRM.) In contrast, in the LAGAN alignment, the rightmost two positions of the site are not aligned to each other, with the implication that approaches that look for orthologous binding sites in a fixed alignment will fail to find this orthologous pair. These two sites are not completely aligned by LAGAN with any tested setting of the gap penalty (unpublished data). The same scenario is observed for the eve stripe 1 enhancers, with a pair of bicoid sites (Figure 7, top right). (Bicoid is a known regulator of this CRM.) Note that in both examples, the alternative alignment found by the motif-agnostic method (LAGAN) is better than or as good as the Morphalign method in terms of number of matches, suggesting that this is going to be a common case of alignment “error,” especially when analyzing binding site evolution.

The next snapshot (Figure 7, bottom), from the “stumps_hbr_early” CRM in the MESODERM set, shows another merit of the Morphalign output: a binding site (for the Dorsal transcription factor) that is present in one species and absent in the other, yet is only partly aligned between the species. Here, the MORPH framework has apparently considered (1) alignments in which the entire site is unaligned and (2) alignments in which the site is not predicted, but its first two positions align between the two species. The final alignment output by the Morphalign program is an “average” over both types of alignments. This leads to the interesting scenario of a binding site overlapping an aligned-block boundary. Looking for binding sites entirely in aligned blocks or entirely outside, as is done by the Stubb program for example, would not reveal this site.

### Conclusions and Future Work

We have presented a novel probabilistic framework for two-species CRM prediction and analysis, combining the established probabilistic (HMM-based) approaches to two distinct problems: sequence alignment and CRM analysis. Our implementation of this framework is available as source code, and will particularly help researchers studying *cis-*regulatory evolution. We have used synthetic data to showcase the potential advantages of the new framework, in improving alignment as well as binding site prediction accuracy. We have demonstrated that CRM prediction is greatly improved with the new method over existing methods that use two-species data. We present the results of using our new motif-aware alignment tool on two well-established regulatory networks in *Drosophila*. These are publicly available via a Web interface. We have used these alignments to demonstrate that the new framework highlights ambiguities in the alignments, and produces alignments that are significantly different from those using a traditional alignment program like LAGAN, or even a motif-agnostic version of the same probabilistic framework. Finally, we have demonstrated that our proposed method paints a very different picture of binding site evolution, namely, one with significantly less loss or gain of functionality among aligned binding sites than projected by motif-agnostic methods. We have showcased specific examples of why this difference arises, and found the common source to be local misalignments by the traditional methods, which are not aware of the locations of potential binding sites. It is easy to appreciate that such traditional methods will always make arbitrary decisions when there are two alternative alignments with the same score, and if a pair of orthologous binding sites falls at the positions of alignment ambiguity, they may be misaligned. When we use motif information, our decision is no longer arbitrary (e.g., the example of Figure 7, top right panel), especially in light of the common opinion that a good binding site tends to be conserved evolutionarily.

The Insert and Delete states of the MORPH model are somewhat misleading in their names. These states emit not only the evolutionary insertions and deletions that happened between the species, but also the orthologous regions that are so diverged that they are better left unaligned. It may be beneficial to model these two types of sequences (indels and orthologous but highly diverged) separately, since they may have distinct statistical properties. This is an interesting direction for future work.

It is worth discussing here the two LLR scores (LLR1 and LLR2) reported by the MorphMS program. The LLR2 score contrasts the MORPH model with a null model in which the sequences are assumed unrelated, and thus implements a “homology testing” approach. This is akin to methods designed for identifying conserved noncoding sequences, such as phastCons [35] and Regulatory Potential scores [36], the difference being that MORPH explicitly accounts for binding site occurrences in computing its LLR2 score. Admittedly, in the tests discussed in “Computational Prediction of a Large Class of CRMs” in Results/Discussion, in which a large collection of 53 motifs was used for CRM prediction, the distinction between LLR2 and these other scores may be somewhat blurred; the distinction will be more pronounced in the tests of “Alignment and Regulatory Evolution in Two Specific Pathways” in Results/Discussion where smaller, pathway-specific sets of motifs were used. On a related note, we would like to point out that a possible way to use the MorphMS scores would be to use the LLR2 score to identify the highly conserved regions of the genome, and then use the LLR1 score on these to identify putative CRMs that have a significant clustering of binding sites in them.

An obvious line of future research is to combine pairwise alignments into a multiple alignment of three or more species. We may adopt ideas of consistency-based clustering [13] in merging the marginal alignment probabilities from separate pairwise alignments. Motif-based multiple alignments of CRMs from the several *Drosophila* genomes will prove to be an invaluable resource for studies on binding site turnover, such as the recent work of Moses et al. [28]. This, in turn, will be crucial to our understanding of how regulatory sequences evolve, and in computational prediction of additional regulatory sequences using comparative genomics.

## Materials and Methods

### Model.

We first describe the probabilistic process (MORPH model) for generation of two orthologous CRMs using given PWM motifs.

**Figure 8:**
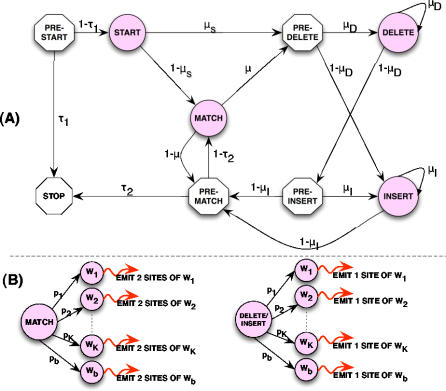
Hidden Markov Model Structure of the MORPH Model (A): Transition probabilities among various states. Circular states have emissions, and octagonal states do not. (B) Motif emissions from Match, Insert, and Delete states. For each, one of *K* motif states, or the background motif state is visited. In case of the Match state, two aligned sites are emitted, whereas for the Insert and Delete states, only one unaligned site is emitted.

*Alignment states.* The main states of the model are Start, Stop, Match, Delete, and Insert. Of these, the first two, i.e., Start and Stop states, have no emissions, the Match state emits two strings of the same length, the Delete state emits a string to be appended only to S_1_, and the Insert state emits a string to be appended only to S_2_. For clarity of exposition, we illustrate the HMM as in Figure 8A, with four additional non-emission states “Pre-Start,” “Pre-Match,” “Pre-Delete,” and “Pre-Insert.” All allowed transitions and their probabilities are shown in Figure 8A. Semantics of the nine states are explained in Table 1. We note that the alignment is generated by alternating between the Match state and the Delete/Insert states. That is, the generated alignment will have blocks of aligned positions, separated by unaligned strings in either or both species. The model does not make any further discrimination of how the unaligned string between two successive aligned blocks were formed. The interblock unaligned string in the first species is generated, followed by that in the second species. Hence, there is a transition from the Delete to the Insert state (via Pre-Insert), but the reverse transition is not allowed.

**Table 1:**
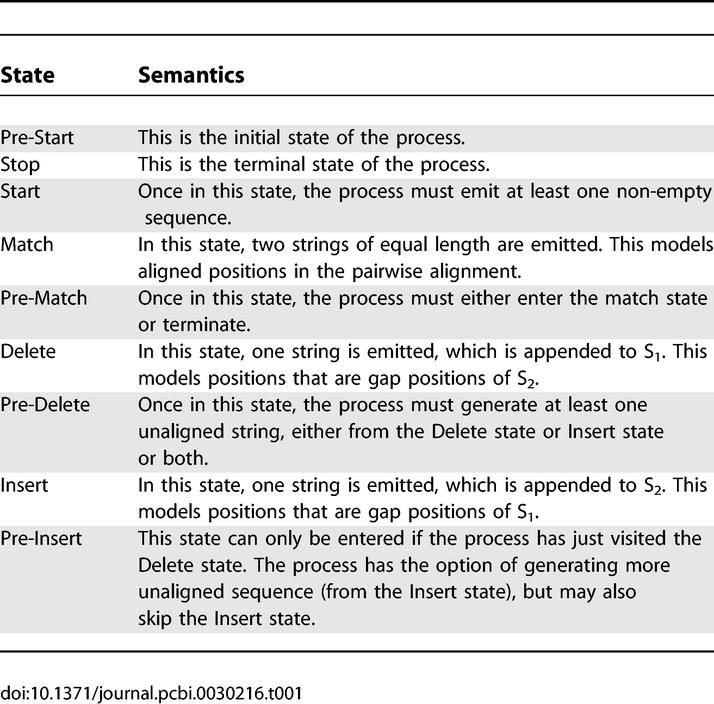
Semantics of the States of the HMM

The edges in Figure 8A show the probabilities associated with the respective state transitions. Thus, the Match state has a μ probability of exiting to Pre-Delete and generating unaligned sequence in at least one species. The Delete state has μ_D_ probability of continuing generation of unaligned sequence in *S*_1_, whereas the Insert state has μ_I_ probability of staying put. From the Start state, there is a μ_S_ probability that unaligned sequences will be generated before the first aligned block is formed. τ_2_ is the termination probability from the Pre-Match state. We can now form the transition probability matrix for the main five states of the HMM, **S**tart, S**T**op, **M**atch, **D**elete, and **I**nsert, as shown in Table 2. τ_1_, τ_2_, μ_S_, μ_D_, μ_I_, and μ are parameters of the model that will be trained from the data, as described later.

**Table 2:**
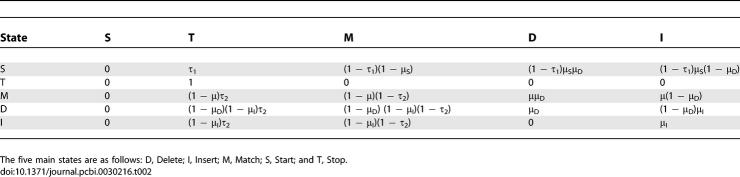
Transition Probabilities for Alignment States in the MORPH Model

*Motif states and emission probabilities.* Once in a Match, Delete, or Insert state, the process transitions to one of several available “motif” states in order to decide which string to emit (Figure 8B). The available states include one state for each of the *K* input PWMs *W*_i_, as well as a “background state” *W*_b_ corresponding to nonbinding sites of unit length. All states are named by the motifs they represent: {*W*_1_, *W*_2,_ …, *W*_K_, *W*_b_}, and are called the motif states. Each state has a fixed probability *p*_i_ of being chosen, called its motif transition probability. Each state has its own emission probability distribution, determined by the PWM *W*_i_ that the state represents. (The Background state represents a single-column PWM capturing background nucleotide frequencies.) For motif states in Delete or Insert states, the emission probability distribution is directly prescribed by the respective PWM: the probability that state *W* emits a string *s* of length *l* (where *l* must be the length of PWM *W*) is 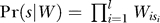 . Motif states in the Match state emit two equal-length strings related by a stochastic model of binding site evolution. (This is true also of the Background motif state, if visited while in the Match state.) The probability that state *W* emits strings *s* and *t*, each of length *l* (where *l* must be the length of PWM *W*) is given by:

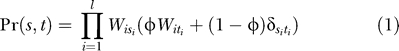
where δ*_jk_* is the Kronecker delta and *φ* is the neutral substitution probability between the two species. This model was used in our earlier work [9,37] and is a special case of the Felsenstein 81 model [38] with equilibrium frequencies given by the PWM *W*.

The motif transition probabilities {*p_1_*, *p_2_*,…, *p_K_*, *p_b_*} are parameters of the model, with the constraint 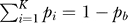 , and are inferred from the data. The evolutionary parameter *φ* in Equation 1 is related to the expected conservation level in aligned sites. For an aligned position whose emission probabilities in each species are given by the probability distribution *f*_α_, we expect (from Equation 1) 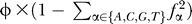 mismatches per position. Although Equation 1, as used in previous publications, interprets *φ* as a neutral substitution probability, here it is the effective neutral substitution probability, conditional on the fact that the site survived during evolution and was therefore aligned. We prefer to interpret *φ* simply as a parameter that controls what fraction of aligned positions are expected to be conserved.

### Algorithm and implementation.

We employ rigorous maximum likelihood estimation of the model parameters τ_1_, τ_2_, μ_S_, μ_D_, μ_I_, μ, and all *p*_i_'s. That is, the algorithm attempts to learn the values of these parameters, collectively referred to as Θ, so as to maximize the probability of generating the sequence data *S* given Θ. (The algorithm used finds local maxima of the likelihood; see below.) Let *T* be a particular “path” in the generative process, i.e., a sequence of states that were visited in the generation of *S. T* is “hidden” information, hence the likelihood has to be computed by summing over all possible *T*. In other words, we have to find Θ that maximizes 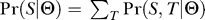 . (The PWMs {*W*_1_, *W*_2_,…, *W*_K_, *W*_b_} are known parameters of the model, and are left out of this expression for clarity.) We use an E-M approach to maximize Pr(*S*|Θ). This is an iterative update algorithm that is guaranteed to improve the likelihood Pr(*S*|Θ) in every iteration, until convergence to a local optimum. In our case, the E-M strategy is implemented by adapting the popular Baum-Welch algorithm for HMMs [14,39]. Adapting the original Baum-Welch algorithm to our probabilistic model involves considerable reformulation, and these calculations are omitted here for clarity. Our algorithm belongs to the general algorithmic paradigm of “dynamic programming,” akin to Needleman-Wunsch alignment, and has quadratic time complexity. In particular, its running time is O(*L* ^2^ *Kl*_max_), where *L* is the length of the sequence(s), *K* is the number of input PWMs, and *l*_max_ is the length of the longest PWM.

Our implementation of the MORPH probabilistic framework allows some additional features that the user may find useful.

*Background.* The user may specify separate background sequences for the two species, for the purpose of training the background motif *W*_b_.This is motivated by the common observation that orthologous genes may have very different nucleotide composition in their respective regulatory regions. If this option is exercised, the evolutionary model specified by Equation 1 (for background positions) is no longer time reversible. The joint probability of seeing base *s* in species 1 and base *t* in species 2 is now given by 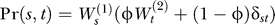 , where *W* ^(1)^ and *W* ^(2)^ are the background PWMs in the two species. Also, the user may specify the “Markov order” of the background, in order to capture neighboring nucleotide dependencies in typical sequences. For example, a first-order Markov background captures dinucleotide frequencies.

*Special Match state.* The user has the option of specifying that the Match state only emit non-background sites, i.e., when in the Match state, the motif transition probability for *W*_b_ is zero. For other PWMs, motif transition probabilities (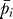 ) in the Match state are scaled versions of their respective values for the Insert and Delete states. That is, 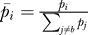 . This option may be useful when analyzing highly diverged species in which one expects only binding sites to be conserved and alignable.

*Speedup.* The E-M parameter estimation algorithm has quadratic time complexity because it considers the possibility of every position *i* in the first sequence being aligned with every position *j* in the second sequence. The user may opt to have the algorithm consider only those pairs of positions that are not too “far removed,” e.g., |*i* − *j*| < 100. This option leads to a significant speedup of the algorithm, with a negligible effect on accuracy. This is similar to the idea of band alignment [40].

*Estimation of the divergence parameter.* By default, the evolutionary parameter *φ*, controlling the expected conservation level at aligned sites (see “Motif States and Emission Probabilities” in Materials and Methods), is user-specified. (The user may have an estimated value of the neutral substitution probability based on existing packages such as PAML [25].) We provide the option of having this parameter heuristically estimated, by starting with the default value, calculating the conservation level of aligned positions given the data, and adjusting the *φ* parameter based on these calculated conservation levels.

The current implementation aligns a typical CRM of approximate length 1,000 in a few minutes, running on a single processor workstation.

### MorphMS.

The MorphMS program is designed for CRM prediction from two-species data. Given two orthologous sequences, it scans one of the sequences with a fixed-length window (in fixed-length shifts), and for each such window, computes the boundaries of its orthologous window in the other sequence by using a standard alignment tool. Thus, a “hard” alignment is used in this first step of detecting “orthologous window pairs,” after which the alignment information is discarded. For each window pair, MorphMS uses the MORPH model to compute the maximum likelihood parameters Θ*_m_*. It then computes two LLRs, by comparing this maximum likelihood Pr(*S*|Θ*_m_*) to the likelihood under two suitable null models.

In the first score, called LLR1, the null model is the maximum likelihood model under the constraint that all motif transition probabilities *p*_i_, except that for the background, are zero. That is, the first null model is the maximum likelihood model in the absence of motifs. The score LLR1 therefore captures whether we can better explain the data by allowing it to contain binding sites for the given transcription factors.

In the second score, called LLR2, the null model includes all motif transition probabilities as learnable parameters, but requires that the Match state is not visited, i.e., the entire data is generated by a single visit to the Delete state, followed by a single visit to the Insert state. The LLR2 score measures how much of an improvement (to the likelihood) comes from allowing the data to contain aligned sites. For example, a window pair that was erroneously marked as orthologous, and in reality consists of two completely unrelated sequences each containing binding site clusters, will not receive a high LLR2 score.

In summary, the LLR1 score accounts for binding sites, whereas LLR2 accounts for alignment. The MorphMS program reports the LLR1 and LLR2 scores for every window pair, and leaves the analysis of these scores to the user. Each of these scores, being an LLR, is comparable across window pairs, implying that the user may choose the top scoring window pairs as CRM predictions.

### Morphalign.

Morphalign is a pairwise alignment program for orthologous CRMs that uses known PWM motifs and the MORPH probabilistic framework. It is very similar to the MorphMS program, except that it processes the entire input sequence instead of sliding a window, and produces a graphical output at the end. It uses potential binding sites to impose a higher-order structure on the sequences and performs alignment on this higher-order structure. All possible parses of each sequence into their higher-order structure are considered and weighted probabilistically, as described in “Alignment and Regulatory Evolution in Two Specific Pathways” in Results/Discussion. Moreover, in contradistinction to usual alignment methods, Morphalign highlights the ambiguous parts of the alignment and quantifies them, thus providing a more complete picture of alignment of two moderately diverged sequences.

The Morphalign program comes with a graphical visualization tool that produces an HTML file (with embedded JavaScript code) that may be viewed through any browser, making the visualization highly portable. The most likely alignment (Viterbi solution of the HMM in the MORPH framework) or the maximum expected accuracy alignment (explained below) forms the backbone of the display, and is displayed in the usual alignment format with gaps and nucleotides. For any pair of positions (in the two sequences), their marginal alignment probability is shown (if above some threshold) by a colored line whose thickness is proportional to the probability. This allows nonaligned positions (in the backbone alignment) to be flagged as being potentially orthologous. The alignment display is annotated by presence of potential binding sites. The marginal probability of a binding site for any particular transcription factor at any particular position is shown by colored (blue) bars at the appropriate position, as shown in Figure 5.

The user has two options regarding how to display the backbone of the alignment (i.e., which position is shown vertically aligned with which position). The Viterbi option uses the maximum likelihood path through the HMM and the Match states that this path goes through, to decide this backbone. Another option, which we have found useful in practice, is to vertically line up all those pairs of positions whose marginal alignment probabilities have the largest sum. This is called the maximum expected accuracy alignment [12].

### Data and experiments.

*Synthetic data experiments.* The MORPH probabilistic process was sampled from, with parameter values τ_1_ = 0.005, τ_2_ = 0.005, and *φ* = 0.5. This resulted in sequences of median length 487 bp. PWMs corresponding to *Drosophila* transcription factors Bicoid, Hunchback, Knirps, Kruppel, Tailless, and Caudal and the TorRE binding factor were used in the sequence generation process. (PWM lengths varied between 9 and 15.) LAGAN was run with its default parameters, as was done in the evaluations performed by Pollard et al. [41]. Morphalign and Stubb were run with the correct value of the evolutionary parameter *φ* = 0.5. Each of these two programs outputs, for every position in a sequence, a “marginal probability” that an occurrence of a binding site begins at that position. We used a threshold of 0.1 on this marginal probability to predict binding sites.

*D. melanogaster and D. mojavensis comparisons.* We started with a complete list of 284 nonredundant (and nonoverlapping) CRMs from the REDfly database [27], and retained only those 208 cases for which the CRMs (with their 1-Kbp flank on either side) had clear orthologs in D. mojavensis. We used a set of 53 PWMs based on the FlyReg database [24] and the online resource maintained by Pollard (http://rana.lbl.gov/~dan/matrices.html). These were all motifs that were based on at least five verified binding sites from the FlyReg database.

Short tandem repeats in CRMs were predicted by running the Tandem Repeat Finder (TRF) program of Benson [34] with parameters: match = 2, mismatch = 3, indel = 5, match probability = 0.8, indel probability = 0.1, minimum score = 25, and maximum period = 10. This finds approximate tandem repeats (with mismatches and indels) of periodicity up to 10.

All runs of Morphalign on the real datasets were done so as to output the maximum expected accuracy alignments (see ”Morphalign” in Materials and Methods), rather than the maximum likelihood (Viterbi) alignment.

Binding sites for the Bicoid transcription factor (“Morphalign Presents a Different Picture of Binding Site Loss and Gain” in Results/Discussion) were obtained as follows: the match score of any string to the PWM was computed as usual by comparing the probability of sampling the string from the PWM to the probability of sampling it from background, i.e., the LLR score of the string. (The background used was constructed from the nucleotide frequencies of the CRM in which the string is located.) The maximum possible LLR score for any string was computed, and all strings with an LLR score at least 50% of this maximum were considered as binding sites for Bicoid.

## Abbreviations

CRM: *cis*-regulatory module
E-M: Expectation-Maximization
HMM: Hidden Markov model
LLR: log-likelihood ratio
PID: percentage identity
PWM: position weight matrix

## References

[pcbi-0030216-b001] Needleman SB, Wunsch CD (1970). A general method applicable to the search for similarities in the amino acid sequence of two proteins. J Mol Biol.

[pcbi-0030216-b002] Altschul SF, Gish W, Miller W, Myers EW, Lipman DJ (1990). Basic local alignment search tool. J Mol Biol.

[pcbi-0030216-b003] Schroeder MD, Pearce M, Fak J, Fan H, Unnerstall U (2004). Transcriptional control in the segmentation gene network of Drosophila. PLoS Biol.

[pcbi-0030216-b004] Davidson EH, Rast JP, Oliveri P, Ransick A, Calestani C (2002). A genomic regulatory network for development. Science.

[pcbi-0030216-b005] Berman BP, Nibu Y, Pfeiffer BD, Tomancak P, Celniker SE (2002). Exploiting transcription factor binding site clustering to identify cis-regulatory modules involved in pattern formation in the Drosophila genome. Proc Natl Acad Sci U S A.

[pcbi-0030216-b006] Halfon MS, Grad Y, Church GM, Michelson AM (2002). Computation-based discovery of related transcriptional regulatory modules and motifs using an experimentally validated combinatorial model. Genome Res.

[pcbi-0030216-b007] Frith MC, Hansen U, Weng Z (2001). Detection of cis-element clusters in higher eukaryotic DNA. Bioinformatics.

[pcbi-0030216-b008] Rajewsky N, Vergassola M, Gaul U, Siggia ED (2002). Computational detection of genomic cis-regulatory modules applied to body patterning in the early Drosophila embryo. BMC Bioinformatics.

[pcbi-0030216-b009] Sinha S, van Nimwegen E, Siggia ED (2003). A probabilistic method to detect regulatory modules. Bioinformatics.

[pcbi-0030216-b010] Brudno M, Do CB, Cooper GM, Kim MF, Davydov E (2003). LAGAN and Multi-LAGAN: efficient tools for large-scale multiple alignment of genomic DNA. Genome Res.

[pcbi-0030216-b011] Batzoglou S (2005). The many faces of sequence alignment. Brief Bioinform.

[pcbi-0030216-b012] Holmes I, Durbin R (1998). Dynamic programming alignment accuracy. J Comp Biol.

[pcbi-0030216-b013] Do CB, Mahabhashyam MS, Brudno M, Batzoglou S (2005). ProbCons: probabilistic consistency-based multiple sequence alignment. Genome Res.

[pcbi-0030216-b014] Durbin R, Eddy S, Krogh A, Mitchison G (1998). Biological sequence analysis: probabilistic models of proteins and nucleic acids.

[pcbi-0030216-b015] Thorne JL, Kishino H, Felsenstein J (1991). An evolutionary model for maximum likelihood alignment of DNA sequence. J Mol Evol.

[pcbi-0030216-b016] Holmes I, Bruno WJ (2001). Evolutionary HMMs: a Bayesian approach to multiple alignment. Bioinformatics.

[pcbi-0030216-b017] Knudsen B, Miyamoto MM (2003). Sequence alignments and pair hidden Markov models using evolutionary history. J Mol Biol.

[pcbi-0030216-b018] Wang J, Keightley PD, Johnson T (2006). MCALIGN2: faster, accurate global pairwise alignment of non-coding DNA sequences based on explicit models of indel evolution. BMC Bioinformatics.

[pcbi-0030216-b019] Berezikov E, Guryev V, Plasterk RHA, Cuppen E (2004). CONREAL: conserved regulatory elements anchored alignment algorithm for identification of transcription factor binding sites by phylogenetic footprinting. Genome Res.

[pcbi-0030216-b020] Hallikas O, Palin K, Sinjushina N, Rautiainen R, Partanen J (2006). Genome-wide prediction of mammalian enhancers based on analysis of transcription-factor binding affinity. Cell.

[pcbi-0030216-b021] Stormo GD (2000). DNA binding sites: representation and discovery. Bioinformatics.

[pcbi-0030216-b022] Wingender E, Chen X, Fricke E, Geffers R, Hehl R (2001). The TRANSFAC system on gene expression regulation. Nucleic Acids Res.

[pcbi-0030216-b023] Vlieghe D, Sandelin A, Bleser PJD, Vleminckx K, Wasserman WW (2006). A new generation of JASPAR, the open-access repository for transcription factor binding site profiles. Nucleic Acids Res.

[pcbi-0030216-b024] Bergman CM, Carlson JW, Celniker SE (2005). Drosophila DNase I footprint database: a systematic genome annotation of transcription factor binding sites in the fruitfly, Drosophila melanogaster. Bioinformatics.

[pcbi-0030216-b025] Yang Z (2007). PAML 4: phylogenetic analysis by maximum likelihood. Mol Biol Evol.

[pcbi-0030216-b026] Cartwright RA (2005). DNA assembly with gaps (Dawg): simulating sequence evolution. Bioinformatics.

[pcbi-0030216-b027] Gallo SM, Li L, Hu Z, Halfon MS (2006). REDfly: a Regulatory Element Database for Drosophila. Bioinformatics.

[pcbi-0030216-b028] Moses AM, Pollard DA, Nix DA, Iyer VN, Li XY (2006). Large-scale turnover of functional transcription factor binding sites in Drosophila. PLoS Comput Biol.

[pcbi-0030216-b029] Emberly E, Rajewsky N, Siggia ED (2003). Conservation of regulatory elements between two species of Drosophila. BMC Bioinformatics.

[pcbi-0030216-b030] Sinha S, Siggia ED (2005). Sequence turnover and tandem repeats in cis-regulatory modules in Drosophila. Mol Biol Evol.

[pcbi-0030216-b031] Sinha S, Schroeder MD, Unnerstall U, Gaul U, Siggia ED (2004). Cross-species comparison significantly improves genome-wide prediction of cis-regulatory modules in Drosophila. BMC Bioinformatics.

[pcbi-0030216-b032] Grad YH, Roth FP, Halfon MS, Church GM (2004). Prediction of similarly acting cis-regulatory modules by subsequence profiling and comparative genomics in Drosophila melanogaster and D. pseudoobscura. Bioinformatics.

[pcbi-0030216-b033] Zhou Q, Wong WH (2004). CisModule: de novo discovery of cis-regulatory modules by hierarchical mixture modeling. Proc Natl Acad Sci U S A.

[pcbi-0030216-b034] Benson G (1999). Tandem repeats finder: a program to analyze DNA sequences. Nucleic Acids Res.

[pcbi-0030216-b035] Siepel A, Haussler D (2004). Combining phylogenetic and hidden Markov models in biosequence analysis. J Comput Biol.

[pcbi-0030216-b036] Elnitski L, Hardison RC, Li J, Yang S, Kolbe D (2003). Distinguishing regulatory DNA from neutral sites. Genome Res.

[pcbi-0030216-b037] Sinha S, Blanchette M, Tompa M (2004). PhyME: a probabilistic algorithm for finding motifs in sets of orthologous sequences. BMC Bioinformatics.

[pcbi-0030216-b038] Felsenstein J (1981). Evolutionary trees from DNA sequences: a maximum likelihood approach. J Mol Evol.

[pcbi-0030216-b039] Baum LE, Petrie T, Soules G, Weiss N (1970). A maximization technique occurring in the statistical analysis of probabilistic functions of Markov chains. Ann Math Statist.

[pcbi-0030216-b040] Chao KM, Pearson WR, Miller W (1992). Aligning two sequences within a specified diagonal band. Comput Appl Biosci.

[pcbi-0030216-b041] Pollard DA, Moses AM, Iyer VN, Eisen MB (2006). Detecting the limits of regulatory element conservation and divergence estimation using pairwise and multiple alignments. BMC Bioinformatics.

